# Gleevec, an Abl Family Inhibitor, Produces a Profound Change in Cell Shape and Migration

**DOI:** 10.1371/journal.pone.0052233

**Published:** 2013-01-02

**Authors:** Zaozao Chen, Elizabeth Lessey, Matthew E. Berginski, Li Cao, Jonathan Li, Xavier Trepat, Michelle Itano, Shawn M. Gomez, Maryna Kapustina, Cai Huang, Keith Burridge, George Truskey, Ken Jacobson

**Affiliations:** 1 Department of Cell Biology and Physiology, University of North Carolina School of Medicine, Chapel Hill, North Carolina, United States of America; 2 Lineberger Comprehensive Cancer Center, University of North Carolina School of Medicine, Chapel Hill, North Carolina, United States of America; 3 Program in Molecular and Cellular Biophysics, University of North Carolina School of Medicine, Chapel Hill, North Carolina, United States of America; 4 Department of Biomedical Engineering, University of North Carolina, Chapel Hill, North Carolina, United States of America; 5 Department of Biomedical Engineering, Duke University, Durham, North Carolina, United States of America; 6 Institute for Bioengineering of Catalonia, University of Barcelona, and Ciber Enfermedades Respiratorias, Barcelona, Spain; 7 Markey Cancer Center and Department of Molecular & Biomedical Pharmacology, University of Kentucky, Lexington, Kentucky, United States of America; 8 Department of Computer Science, University of North Carolina at Chapel Hill, Chapell Hill, North Carolina, United States of America; 9 Department of Pharmacology, University of North Carolina School of Medicine, Chapel Hill, North Carolina, United States of America; Georgia Health Sciences University, United States of America

## Abstract

The issue of how contractility and adhesion are related to cell shape and migration pattern remains largely unresolved. In this paper we report that Gleevec (Imatinib**)**, an Abl family kinase inhibitor, produces a profound change in the shape and migration of rat bladder tumor cells (NBTII) plated on collagen-coated substrates. Cells treated with Gleevec adopt a highly spread D-shape and migrate more rapidly with greater persistence. Accompanying this more spread state is an increase in integrin-mediated adhesion coupled with increases in the size and number of discrete adhesions. In addition, both total internal reflection fluorescence microscopy (TIRFM) and interference reflection microscopy (IRM) revealed a band of small punctate adhesions with rapid turnover near the cell leading margin. These changes led to an increase in global cell-substrate adhesion strength, as assessed by laminar flow experiments. Gleevec-treated cells have greater RhoA activity which, via myosin activation, led to an increase in the magnitude of total traction force applied to the substrate. These chemical and physical alterations upon Gleevec treatment produce the dramatic change in morphology and migration that is observed.

## Introduction

The study of cell migration is essential for understanding a variety of processes including wound repair, immune response and tissue homeostasis; importantly, aberrant cell migration can result in various pathologies [1], [2], [3]. However, the relationship between cytoskeletal dynamics, including actin network growth, contractility, and adhesion, to cell shape and migration remains incompletely understood.

Abl family tyrosine kinases are ubiquitous non-receptor tyrosine kinases (NRTKs) involved in signal transduction [4], [5], [6]. They can interact with other cellular components through multiple functional domains for filamentous and globular actin binding, as well as through binding phosphorylated tyrosines (SH2), polyproline rich regions (SH3), DNA (Abl), and microtubules (Abl Related Gene (Arg)) [7], [8]. Abl family tyrosine kinases have also been found to regulate cell migration [8], [9]. In some cases, Abl family kinases have been reported to promote actin polymerization and migration [10] as well as filopodia formation during cell spreading [11], [12]. By contrast, in other studies Abl was found to restrain lamellipodia extension [13], [14] or inhibit initial cell attachment to the substrate [15]. Abl family kinases have been suggested to regulate cell adhesion size and stress fiber formation [16]; Li and Pendergast recently reported that the Abl family member Arg, could disrupt CrkII-C3G complex formation to reduce β1-integrin related adhesion formation [17]. Thus, a complete understanding of how Abl family kinases regulate cell migration is lacking [8], [9].

In this study, we report that Gleevec (also called Imatinib/STI571), an Abl family kinase inhibitor that is used as a chemo-therapeutic agent for leukemia, produces a profound change in the shape and migration of the rat Nara bladder tumor (NBT-II) cells plated on collagen-coated substrates. Within 20 min of Gleevec treatment the majority of NBT-II cells develop a new D-shaped morphology and start migrating more rapidly and with greater persistence. The new morphology is characterized by stronger cell-substrate adhesion and an increase in the size and number of discrete adhesions which at the leading margin turnover more rapidly. RhoA activity in Gleevec-treated cells was increased which, via myosin activation, led to an increase in the magnitude of total traction forces applied to the substrate. Upon Gleevec treatment, these chemical and physical alterations combined to produce the dramatic change in morphology and migration.

## Results

### Treatment with Gleevec induces a D-shaped morphology in NBTII cells

**Figure 1:**
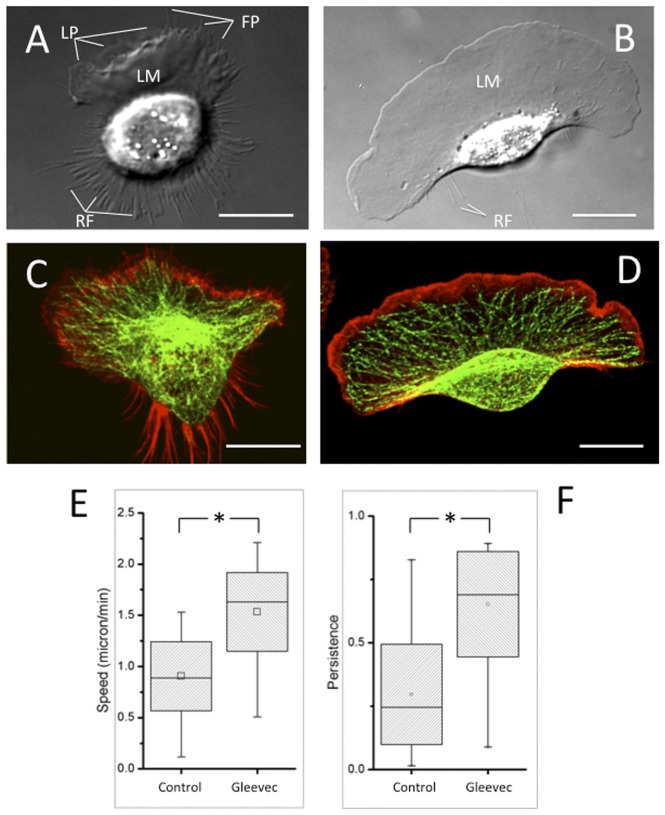
Transformation of NBT-II cells morphology and migratory phenotype after Gleevec treatment. A and B) Representative DIC images of NBT-II cells plated on 10 µg/ml collagen coated substrate. Control cell (A) and cell treated for 30 min with 20 µM Gleevec. (B) Note that a lamellipodial protrusion and a D- (or fan) shaped morphology occurs within 10 minutes of exposure to the Abl-family inhibitor. Lamellae (Marked with “LM”), lamellipodia (Marked with “LP”), filopodia (Marked with “FP”) and retraction fibers (Marked with “RF”) were labeled accordingly. (C and D) Confocal fluorescent images of the f-actin (Rhodamine-phalloidin, Red) and microtubules (alpha-tubulin antibody, Green) in the control (C) and Gleevec-treated cells (D). (E and F) Box and whisker plots of the average cell migration speed (E) and directional persistence (F) for the control group (N = 60) and NBT-II cells treated with 20 µM Gleevec (N = 60). Standard deviations are indicated by the box sizes; maximum and minimum data values are indicated by the extent of the whiskers. The bar and the square inside the box are the median and mean value respectively. Gleevec-treated NBTII cells migrate significantly faster and are more persistent in their directionality (* p<0.001, by students *t*-test). Scale bars are 20 µm.

The morphology of a migrating cell is related to cell migration modes. NBTII is a rat-derived carcinoma cell line [18]. A normal cultured NBTII cell shows typical epithelial morphology; however, when NBTII cells were cultured on type I collagen-coated plastic cell culture dishes for 4–12 h, they acquired a polarized shape and migrate individually, exhibiting an epithelial to mesenchymal transition (EMT) [19], [20], [21], [22]). During our experiments, we observed that NBTII cells on collagen had medium-sized lamellae (Marked with “LM”) and lamellipodia (Marked with “LP”), some filopodia (Marked with “FP”) dynamically formed at the leading edge of the cell, and multiple retraction fibers (Marked with “RF”) formed at the trailing edge of the cell. (**Figure 1A** **, Movie S1**). **Figure 1B** shows NBTII cells cultured on type I collagen for 8 h and then treated with 20 µM Gleevec, an inhibitor of the Abl family of NRTK (Novartis, Stein, Switzerland) for 30 minutes [23], [24]). Within about 10 minutes of Gleevec addition, a profound change in cell morphology can be observed (**Movie S2**). These kinetics can be seen by the change in area of Gleevec-treated cells occurring after about 5 minutes (**Figure S1**). Cells began to form lamellipodial protrusions, which usually merged into a single, intact lamella facing the migration direction (**Movie S3**). This cell morphology may persist for over 8 hours. After Gleevec treatment, cells had reduced numbers of both filopodia and retraction fibers. The actin and microtubule cytoskeleton of NBTII cells or Gleevec-treated NBTII cells differed somewhat (**Figure 1C and 1D**); particularly noticeable were the number of f-actin rich retraction fibers in the control cells. Gleevec-treated NBTII cells had about a 75% increase in migration speed compared with control NBTII cells (**Figure 1E**) and maintained their direction significantly better than control NBTII cells (**Figure 1F**) (**Movie S4**).

**Figure 2:**
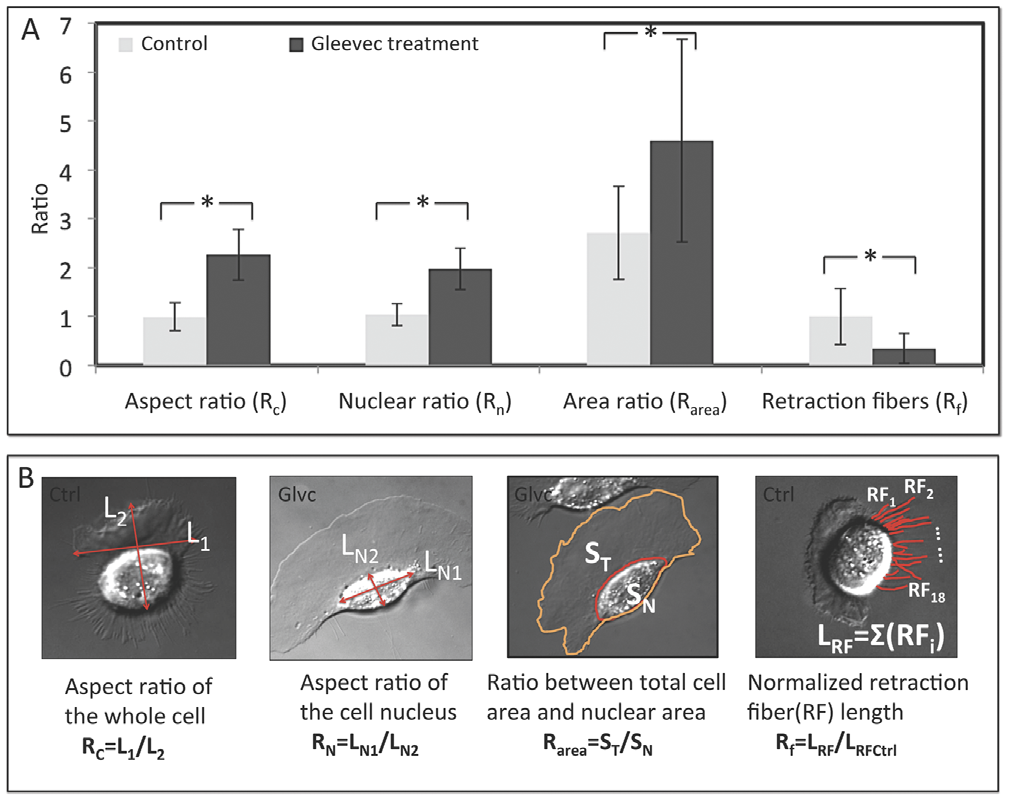
Detailed analysis of cell morphology changes after Gleevec treatment. A) Cell morphology parameters (see text) were analyzed and compared between NBT-II cells from the control group and the group treated with 20 µM Gleevec. B) Schematic figures depicting the calculation of each cell morphology parameter. The 1^st^ and 4^th^ cells from the left, shown in panel B, are samples of control NBT-II cells; while the 2^nd^ and 3^rd^ cells depict NBT-II cells that have been treated with Gleevec. Data are calculated from more than 50 cells for each group. Error bars indicate standard deviations. Control and Gleevec treated cells are significantly different in all four parameters (* indicates p<0.001, by student's *t*-test).

Gleevec treatment induced a pronounced change in cell morphology when compared to control cells (**Figure 1** **and Movies S1, S2, S3**). To better determine the changes in cell morphology, we used four parameters as defined in **Figure 2B**: (1) ***Aspect ratio***, the ratio of the widest dimension of the cell in the direction perpendicular to the direction of migration divided by the longest dimension of the cell in the direction of cell migration; (2) ***Nuclear aspect ratio***, the aspect ratio of the cell nuclear region; (3) ***Area ratio***, the ratio of the total cell area to the nuclear area; and (4) ***Retraction fiber length***, the population average of the sum of the length of all retraction fibers in one individual cell divided by the same parameter calculated for the control cells. The results of our analysis revealed that cells treated with Gleevec had significantly increased aspect ratio, nuclear ratio, and area ratio, while having a reduced retraction fiber length ratio (**Figure 2A**). Interestingly, unlike most known mesenchymal migrating cells, which are typically elongated in the direction of migration, Gleevec-treated cells were elongated in the direction perpendicular to their movement and showed visual similarity to fish or amphibian keratocytes [25], [26], [27] (**Figure 1B**) (**movies 3,4**).

### Both Gleevec concentration and substrate adhesiveness affect NBTII cell migration

**Figure 3:**
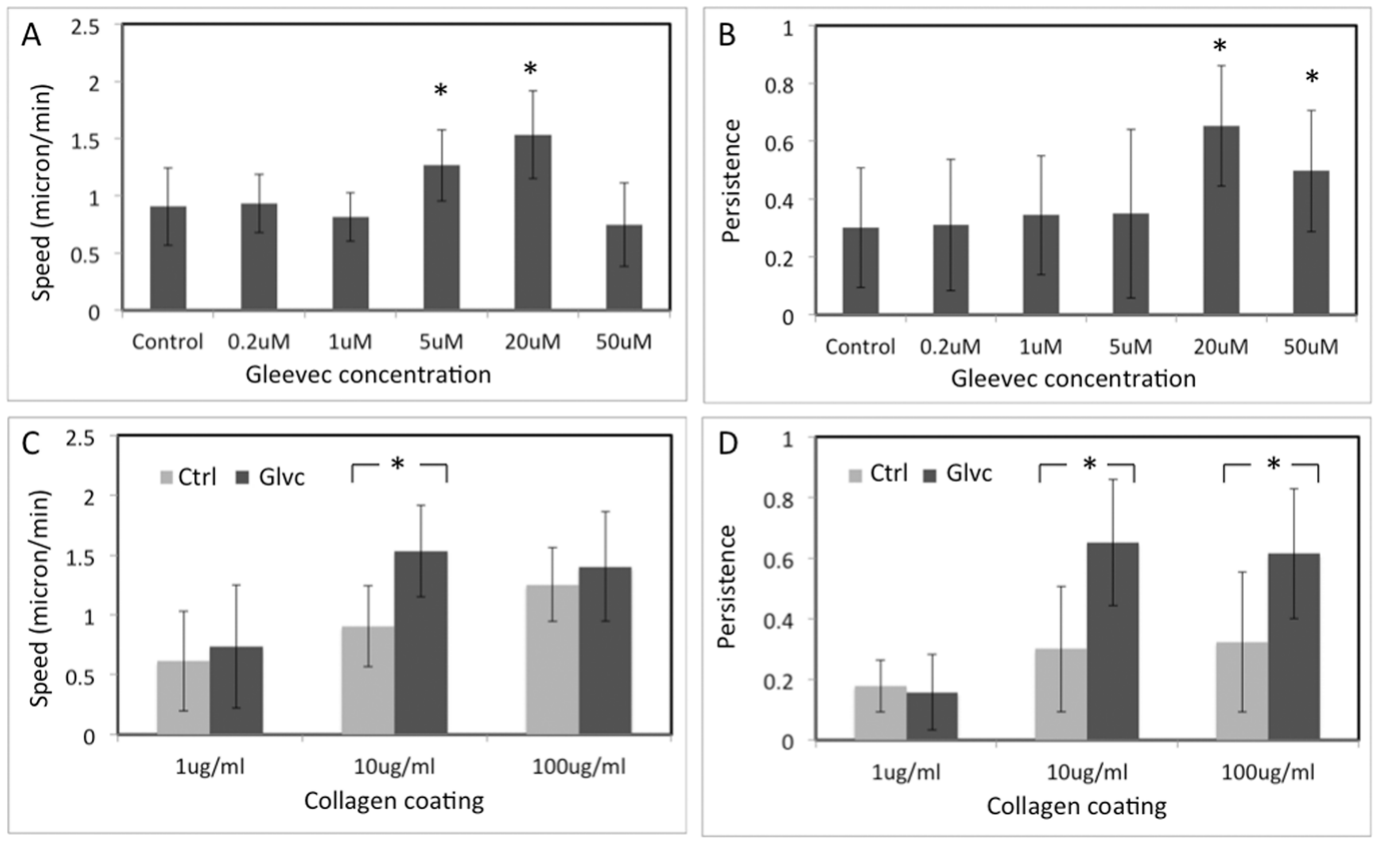
NBTII cell migration behavior depends on substrate adhesiveness and Gleevec concentration. A) and B) Graphs depicting the average cell migration speed (A) and persistence (B) of NBT-II cells from the control group (no inhibitor) and NBT-II cells treated with different concentrations of Abl family kinase inhibitor (Gleevec). Cells were cultured on 10 µg/ml collagen coated substrates. Gleevec concentration has significant effect to both cell migration speed and persistence (ANOVA, p<0.05). Cells treated with 5 µM or 20 µM Abl kinase inhibitor migrated significantly than control cells, while 20 µM and 50 µM group also migrated more persistently than control cells. The significance between control and each Gleevec treated groups was tested by one-way ANOVA followed by Bonferroni's post hoc test, (*) p<0.05. C) and D) Graphs depicting the average cell migration speed (C) and persistence (D) of NBT-II cells on substrates coated with 1 µg/ml, 10 µg/ml, and 100 µg/ml collagen. The average migration speed and persistence of control and Gleevec cells are presented with gray and black bars, respectively. Significance of differences in migration speed and persistence between control and Gleevec treated cells were marked in the figure (* p<0.001, by students *t*-test). For all panels (A–D), results are calculated from more than 30 cells in each group. The error bars indicate standard deviations.

To investigate the NBTII cell dose response for Gleevec concentration we determined migration speed and persistence for NBTII cells treated with different concentrations of Gleevec. Cells were plated on substrates coated with 10 µg/ml collagen. The concentration of Gleevec employed to inhibit Abl family kinase activity was in the range of 0.25 µM to 50 µM. The average cell migration speed reached a maximum (∼2 µm/min), when a 20 µM concentration of Gleevec was used. For lower Gleevec concentrations (0.25 µM, 1 µM), cells did not show a significant speed increase. The highest concentration of Gleevec (50 µM) actually caused cell migration speed to decrease (**Figure 3A**). The ability of NBTII cells to migrate persistently in one direction was also highest after treatment with 20 µM of Gleevec (**Figure 3B**).

To investigate how the substrate adhesiveness influences NBTII cell migration, we tested different concentrations of collagen for substrate coating. With a higher collagen coating concentration, more collagen would absorb to the substrate providing more integrin binding sites, thereby presumably increasing cell-substratum adhesions. For these experiments we used control NBTII cells and cells treated with a 20 µM concentration of Gleevec. For control NBTII cells, when the collagen coating concentration was increased from 1 µg/ml to 100 µg/ml, both the migration speed (**Figure 3C**) and persistence (**Figure 3D**) increased. For the Gleevec-treated NBTII cells, the speed of migration was greatest on the substrates with medium and higher adhesivity (10 and 100 µg/ml collagen). The largest difference in speed and persistence between control and Gleevec-treated NBTII cells occurred at a 20 µM of Gleevec concentration on 10 µg/ml of collagen-coated substrates.

### Gleevec-treated NBTII cells are more adherent to their substrate than control cells

**Figure 4:**
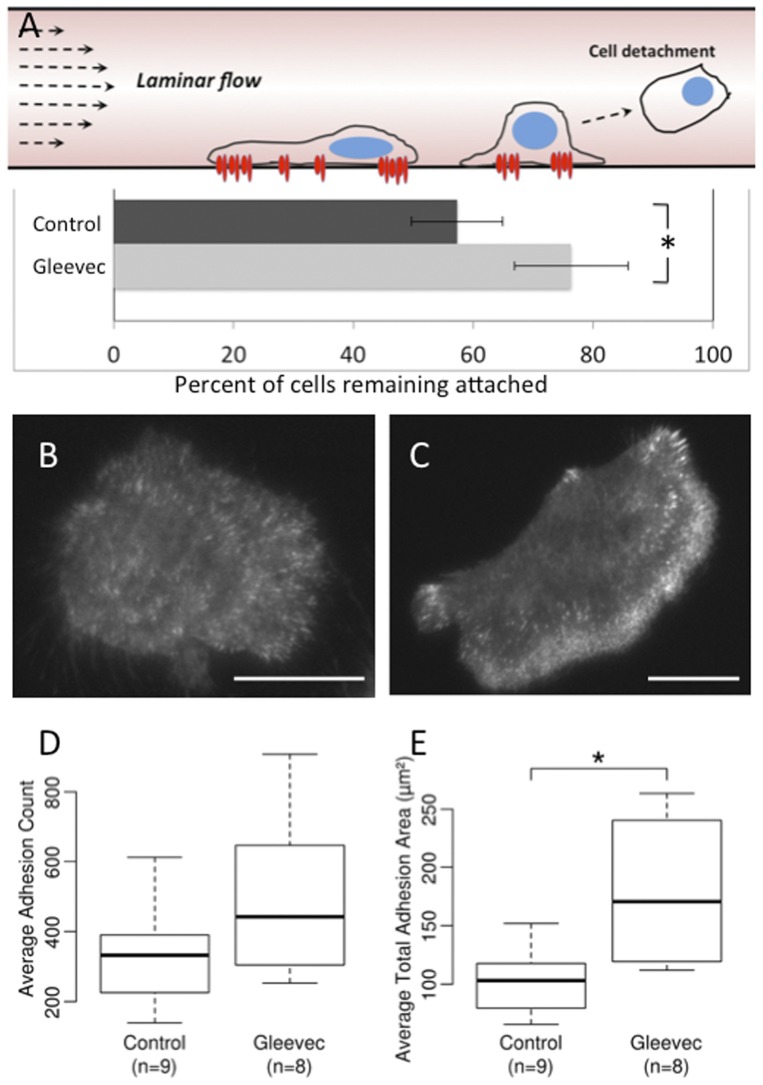
Gleevec-treated cells are more adhesive than control cells. A) A schematic figure (top panel) showing the measurement of cell adhesion strength using a laminar flow system. As the laminar flow rate is increased, more cells detach. Cells were cultured for 4 hours on 10 µg/ml pre-coated collagen Nunc SlideFlask (Thermo) substrates. The fraction of adherent cells remaining after exposure to shear stress of 200 dynes/cm^2^ for 1 min (N = 5; n = 11–20 images per N) is shown in the horizontal bar graph The group of cells treated with 20 µM of Gleevec has significantly higher number of remaining cells after laminar flow exposure (* indicates p<0.05 by the students t-test). B) and C) are representative GFP-Paxillin TIRF images for control NBT-II cells and Gleevec-treated NBT-II cells, respectively. Scale bars are 20 µm. D) and E) The average number of adhesions (D) in control and Gleevec-treated cells and the average total area of the adhesions (E) (* indicates p<0.01 by the students t-test). Error bars indicate standard deviations. Cell counts (n) are listed in the figure.

The highly spread lamellae of Gleevec-treated NBT II cells suggested that they had become more adhesive. Therefore, we investigated cell adhesion strength using a laminar flow system reported previously [28], [29]. Basically, by varying the flow rate, the system generates various shear stresses on cells attached in the flow channel. When applied shear stress exceeds the global cell adhesion strength, cells will detach from the substrate (**Figure 4A**). Images showing the cells attached before and after flow application were recorded and cell numbers were counted (**Materials and Methods** **, Figure S2**). For our experiments, we tested 100 dynes/cm^2^, 200 dynes/cm^2^ and 253 dynes/cm^2^ values of shear stress (**Figure S2G**). We found that a shear stress of 200 dynes/cm^2^ is the most appropriate for estimation of the relative NBTII cell adhesion strength. A shear stress of the 100 dynes/cm^2^ was too weak to affect cell attachment and a shear stress of 253 dynes/cm^2^ quickly removed most of the attached cells. We applied 200 dynes/cm^2^ shear stress for 1 min to the control and Gleevec-treated NBTII cells which were plated four hours before experiment on 10 µg/ml collagen. The fraction of the remaining adherent cells was significantly larger for the cells treated with Gleevec (**Figure 4A**). This result indicates that the global adhesion strength between cells and collagen substrates was increased after inhibition of Abl family kinases. By plotting the fraction of adherent cells remaining after application of shear stress vs. the applied shear stress, we could estimate that critical shear stress at which 50% of the cells detached increased by about 10% (from 214 to 236 dynes/cm^2^) when cells were treated with Gleevec (**Figure S2G**).

We further transfected NBTII cells with GFP-Paxillin to indicate adhesions and employed total internal reflection fluorescence microscopy (TIRFM) to monitor GFP-Paxillin localization on the ventral surface of the cell. Adhesions were automatically tracked and measured by an algorithm developed in a previous report (**Materials and Methods**) [30]. Compared to control NBTII cells (**Figure 4B**), Gleevec-treated cells (**Figure 4C**) had an increased number of adhesions at their leading edge and wings; in addition, the total adhesion number increased by ∼25% (**Figure 4D**) and the total adhesion area increased by ∼70% (**Figure 4E**).

To assess the extent of detachment by bond breakage versus cohesive failure due to membrane rupture, we examined cells after application of sheer stress using confocal microscopy as shown in **Figure S2H–J**. This figure shows that NBT-II cell detachment occurred predominantly at the level of integrin and other adhesion bonds to the matrix coated substratum as opposed to membrane rupture around the adhesion sites. We reached this conclusion because the number of observations of membrane fragments or remnant focal adhesions (**Figure S2J**) were few undetectable indicating that most cells detached by breaking substrate adhesion bonds.

### Punctate adhesions are present at the leading edge of Gleevec-treated D-shape NBT-II cells

**Figure 5:**
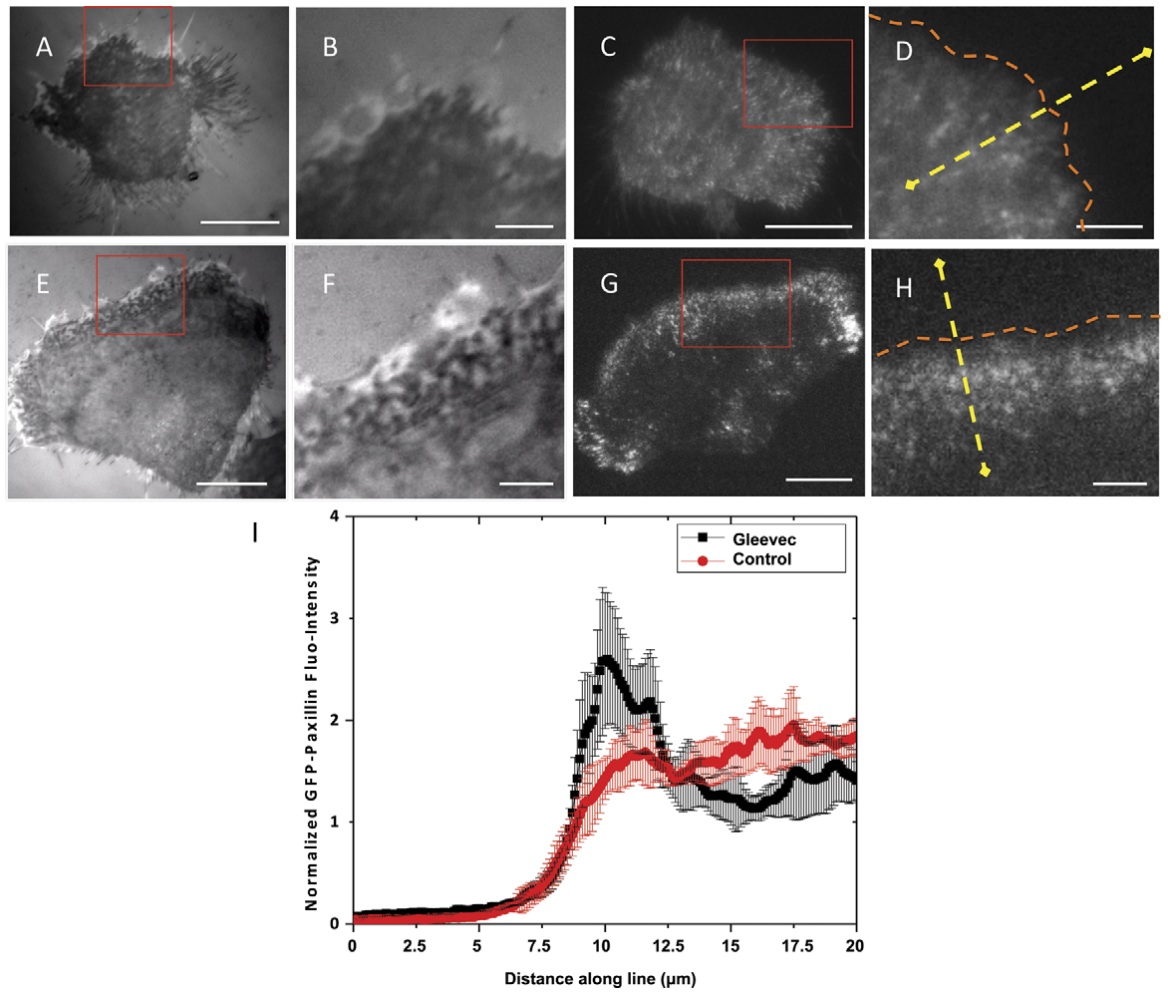
Punctuate adhesions are present at the leading edge of Gleevec-treated NBT-II cells. Panel A) and E) are representative interference reflection images of a migrating control (A) and a Gleevec-treated (E) NBT-II cell, respectively. Dense, dynamic, punctuate adhesions are only observed at the leading edge of the Gleevec-treated cells. Red rectangles on (A) and (E) show the position where thee times magnified images (B) and (F) were taken. Panel C) and G) are representative TIRF images of GFP-Paxillin expressed in control and Gleevec-treated NBT-II cells, respectively. D) and H) Images resulting from amplifying the areas indicated by the red boxes in C) and G) by three times, respectively. Gleevec-treated cells form a band of GFP-Paxillin at the leading edge of the cell. The GFP-Paxillin fluorescent intensity is measured along yellow dotted lines (shown in Figure 5C and 5G) across the leading edge of the cell (4 lines for each cell). I) Multiple cells were used to calculate the distribution of normalized GFP-Paxillin intensity at the leading edge: control NBT-II cells (Black line, n = 12), and D-shaped Gleevec-treated NBT-II cells (red line, n = 12), with the standard deviation shown as gray (for control cells) or pink bars (for Gleevec-treated cells) (detailed calculations are described in Materials and Methods). Gleevec-treated cells NBT-II cells have peak of GFP-Paxillin signal near the leading edge, while control NBT-II cells do not. Scale bars in panels A, C, E and G are 20 µm, and in B, D, F, H are 5 µm. Error bars indicate standard deviations.

TIRFM and Interference Reflection Microscopy were combined to capture time-lapse images of adhesions in migrating NBTII cells. The darker regions in interference reflection image are usually considered regions which are closer to the substrate [31]. In **Figure S3A** and **S3B**, the dark regions in interference reflection images are generally co-localized with the GFP-Paxillin regions in the TIRF image, indicating that those dark regions and dots are actually cell adhesions. The images of the leading edge of control and Gleevec-treated NBTII cells are shown in **Figures 5A** to **5D and 5E to 5H**, respectively. In interference reflection images, the small punctate adhesions were only observed in the leading edge of Gleevec-treated cells (**Figure 5E and 5F**) (**Movie S6**), but not in control cells (**Figure 5A and 5B**) (**Movie S5**). As shown in the TIRF images, compared with control cells (**Figure 5C and 5D**) (**Movie S7**), D- shaped NBTII cells had a larger amount of dotted GFP-Paxillin at their leading edge (**Figure 5G and 5H**) (**Movie S8**). We measured the intensity of GFP-Paxillin along the sample lines indicated in **Figure 5C and 5G**. For each cell, lines crossing the leading edge were summed together and normalized (**Materials and Methods**). We found that D-shaped NBTII cells had significantly increased intensity of GFP-paxillin fluorescence signal in the vicinity of the leading edge (**Figure 5I**). Adhesion turnover in the leading edge of a Gleevec-treated cell was imaged and shown in **Figure S3C or Movie S8**. **Figures S3D-G** are TIRF images of EGFP-Paxillin in Gleevec-treated cells, showing a rim of punctate adhesions at the leading margin as a common feature. The size distribution of punctate adhesions in Gleevec-treated NBTII cells had a peak at ∼350 nm in diameter, and the average area of punctate was 0.1 µm^2^. The dimensions of many punctate adhesions are close to the diffraction limit of the microscope, and some punctates may be even smaller in dimension. Observation of the TIRF movies suggested that these punctate adhesions near the leading edge turned over very rapidly; indeed, intensity analysis [32] indicated an average lifetime of ∼70 s; by contrast, adhesions at the cell wings are much more stable with lifetimes in excess of 5 minutes (**Figure S3H–I**).

### β1 integrin-containing cell adhesions are important for maintaining D-shape morphology and migration status

**Figure 6:**
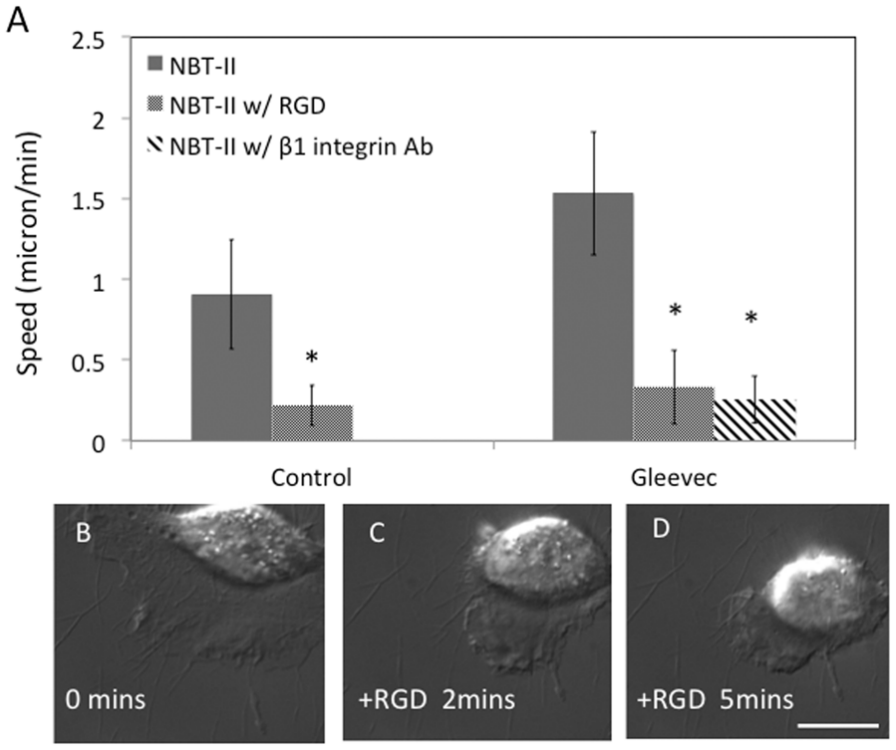
Blocking of integrin related adhesion dramatically inhibits the migration speed of Gleevec-treated NBT-II cells. A) Migration speed of Gleevec-treated cells incubated with either beta1-integrin blocking antibody (1 µg/ml) or an RGD-containing peptide (100 µg/ml). Migration speed was measured 30 minutes after addition of beta1-Integrin blocking antibody or RGD containing peptide (n>10 for each group). Panels B–D) are DIC images of an NBT-II cell treated with Gleevec (20 µM) migrating on a 10 µg/ml collagen-coated substrate, and then treated with 1 µg/ml RGD containing peptide. Images before addition of RGD containing, 2 minutes after and 5 minutes after addition of the RGD peptide are shown. Scale bars are 20 µm. Error bars indicate standard deviations. The statistical significance of difference between control cells with and without RGD, and difference between Gleevec treated cells with and without integrin blocking antibody or RGD is indicated by an (*. p<0.05) as evaluated by one-way ANOVA followed by Bonferroni's post hoc test.

Next, we asked whether integrins were important for the Gleevec induced-phenotype. To competitively disrupt Integrin-collagen binding we employed two different agents: an RGD-containing peptide (Gly-Arg-Gly-Asp-Thr-Pro) [33] and direct β1 integrin blocking antibodies, because β1 integrin is known to bind type I collagen [34], [35], [36]. We found that either 1 µg/ml of β1 integrin blocking antibody or 100 µg/ml RGD-containing peptide (G5646, SIGMA) quickly decreased cell migration speed (**Figure 6A**) and reverted the Gleevec-treated cell morphology approximately back to control NBTII cells (**Figure 6B–D**).

### Gleevec induces changes in actin cytoskeleton, p-MLC localization and traction

**Figure 7:**
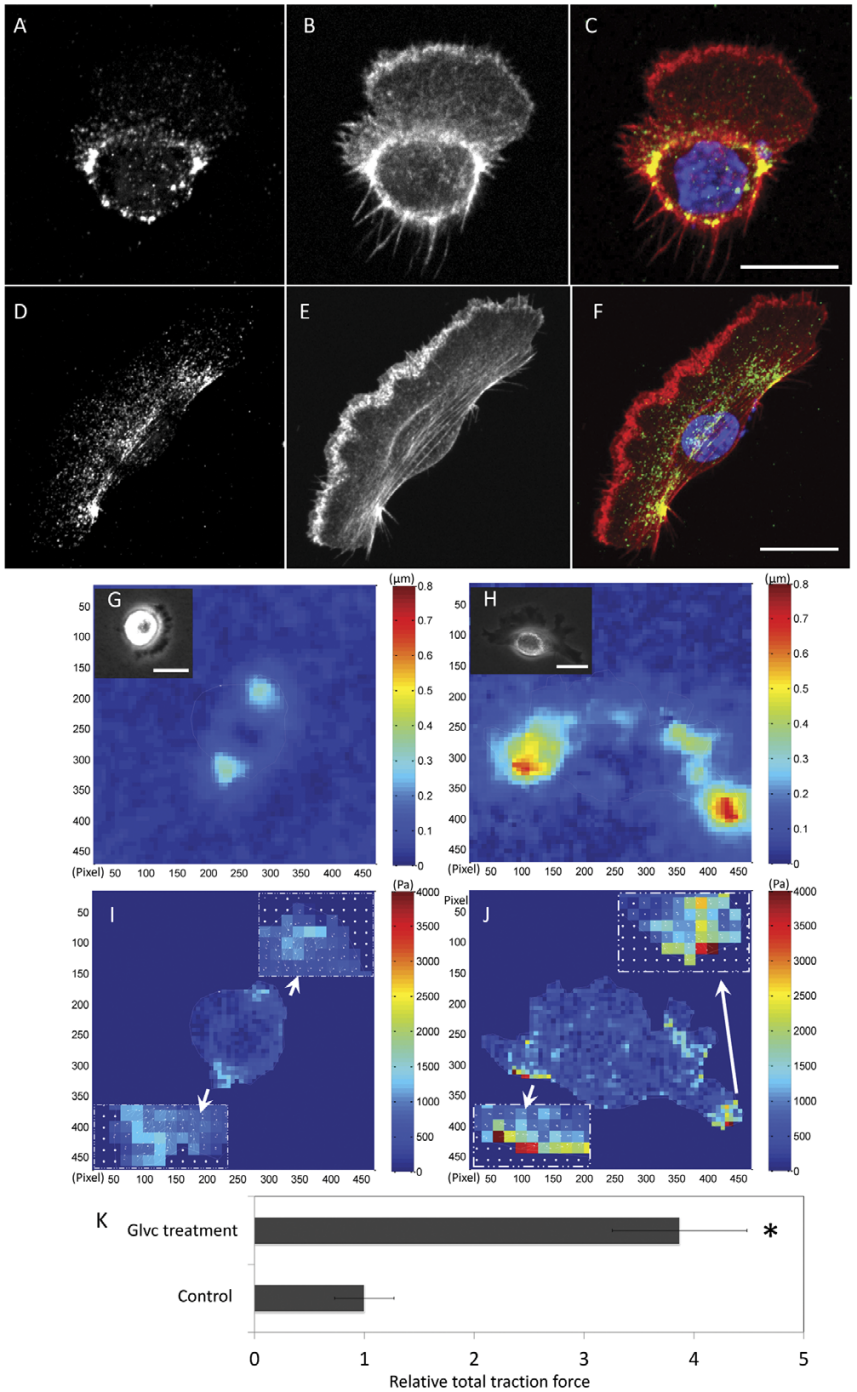
Changes in distribution of active myosin and traction forces after Gleevec treatment. Control NBT-II cells (A–C) or Gleevec-treated cells (D–F) that have been fixed, permeabilized and stained for phosphorylated myosin II light chain (p-MLC) to visualize active myosin localization (A and D) and Rhodamine-phalloidin to visualize the actin cytoskeleton (B and E). (C and F) Overlay images indicate the colocalization of actin bundles and p-MLC (red). The nucleus of the cell is stained with DAPI [60]. Bar = 20 µm. (G to J) Elastic substrate traction mapping of a control NBT-II cell (G and I) and Gleevec-treated NBT-II cell (H–J). (G and H) are the bead displacement maps and (I and J) are the traction maps where color bars indicate relative values (see Methods). The inset images in G and H are the phase image of the control cell and the Gleevec-treated cell. The white lines in G and H are outline of each cell. The inset images of figure I and J are the tractions magnified from indicated cell wings. Panel K is a calculation of the total cell traction force generated by cells (Materials and Methods). The value is normalized to total traction forces from control cells. The bar graph indicates NBTII cells treated with Gleevec generate considerably more total traction force than control NBTII cells. Error bars are standard deviation. Bar = 20 µm. 8 cells were examined for each case. Control and Gleevec-treated cells are significantly different in total cell traction force (* p<0.01, by student's *t*-test).

The filamentous actin and active myosin were detected by fluorescent phalloidin and phosphorylated myosin light chain antibodies, respectively. The distribution of active myosin in control and Gleevec-treated NBTII cells differs. In the case of the control cells, active myosin is localized around the cell nucleus so that some appears near the central part of the trailing edge, with little near the leading edge (**Figure 7A–C**). By contrast, the distribution of active myosin II in Gleevec-treated cells is distributed further away from the nucleus and the rear margin of the cell, mainly at the backside of cell wings, much like keratocytes (**Figure 7D–F**). Since the active myosin status is associated with cell contractility, we compared the traction distribution between the two groups utilizing an elastic substrate methodology [37], [38], [39], [40], [41], [42]. **Figure 7G–H** show the color-coded magnitudes of the bead displacements mapping for control and Gleevec-treated cells, respectively. The white line drawn indicates the outline of the cell. The insets are the phase image of a control (**7G**) or a Gleevec-treated cell (**7H**). **Figure 7I–J** shows the calculated constrained traction map for control and Gleevec-treated cells with the insets showing magnified traction maps at the cell wing regions. Tractions are much higher in the wings of the Gleevec-treated cells than control cells (**Figure 7J**) where immunofluorescence labeling by anti-p-MLC antibody indicates a concentrated region of active myosin proximate to the high traction regions (**Figure 7F**). The Gleevec-treated cells generated a remarkable almost four-fold greater total traction force than control NBTII cells (**Figure 7K**).

### Effects of RhoA family GTPases on D-shaped NBTII cell migration

**Figure 8:**
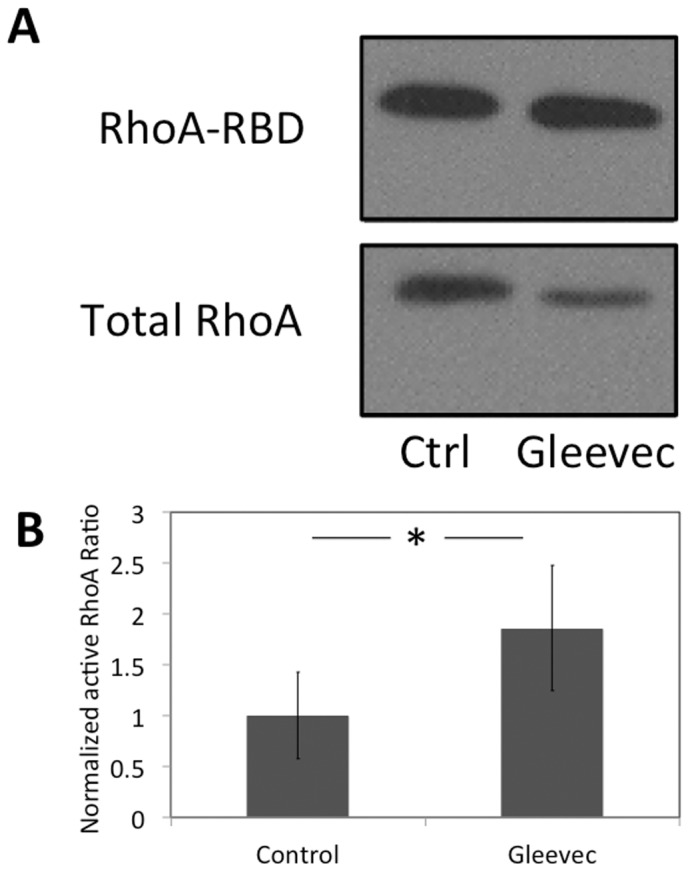
Abl-family kinase inhibition increases RhoA activity. A) A RhoGTPase pull-down assay before and after Gleevec treatment (20 µM). NBT-II cells were cultured on a 10 µg/ml collagen-coated substrate. B) A bar graph quantifying the results from the pull-down assay. (n = 4 experiments). Error bars indicate standard deviations. Control and Gleevec-treated cells are significantly different in RhoA activity (* p<0.05, by student's *t*-test).

The rapid response of NBTII cells to Gleevec suggests that the inhibition of the Abl-family kinases is altering active signaling pathways [8], [43] as opposed to affecting transcriptional regulation. Because of the importance of RhoA GTPase in cell shape and migration [44], [45], [46], we measured its activity before and after Gleevec treatment. Using a RhoA activity pull down assay, we found that RhoA activity significantly increased when Abl-family kinases were inhibited (**Figure 8A**). Cells treated with Gleevec for one hour have nearly doubled RhoA activity when compared to control cells (**Figure 8B**).

**Figure 9:**
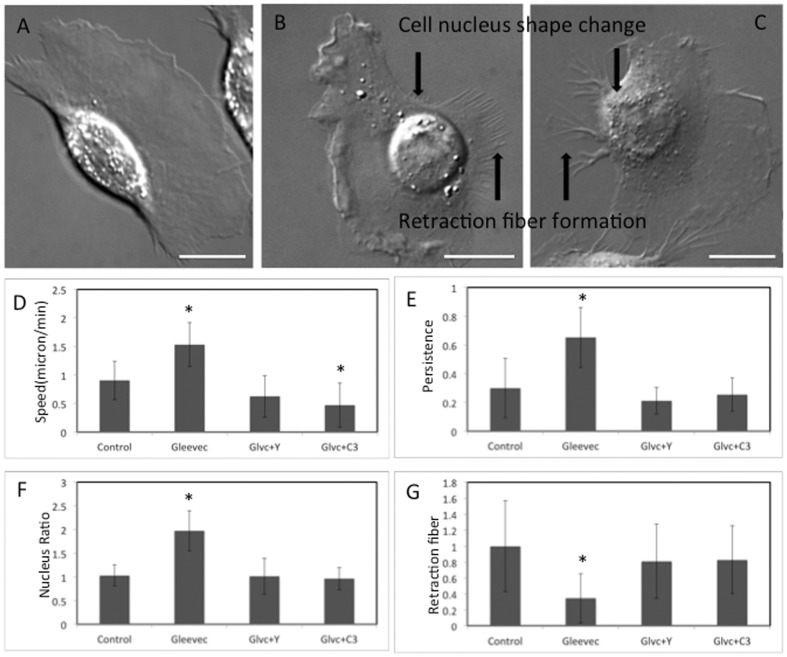
RhoA/ROCK activity is important for the Gleevec phenotype. A), B) and C) are DIC images of NBT-II cell migration status in the presence of 20 uM Gleevec only, both 20 uM Gleevec and ROCK inhibitor(5 uM Y-27632), or both 20 uM Gleevec and RhoA inhibitor (C3, 1 ug/ml), respectively. These panels show that RhoA inhibition after Gleevec treatment increases the number of retraction fibers and produces more rounded nuclei. Scale bars are 20 µm. Panels D) to G) are cell migration speed, cell migration persistence, nuclear aspect ratio and cell retraction fiber ratio, respectively. In each figure, the four bars represent the control group, the 20 uM Gleevec-treated group, the 5 µM Y-27632 +20 µM Gleevec-treated group, and the 1 µg/ml C3 +20 µM Gleevec-treated group, respectively. Error bars indicate standard deviations. At least 15 cells were measured for each group. The significance of the difference between control and other treated groups was evaluated by one-way ANOVA followed by Bonferroni's post hoc test, and marked by (*), p<0.05;.

Because of the significant increase in active RhoA in Gleevec-treated cells, we asked whether RhoA and its downstream effector ROCK were important in the Gleevec-induced phenotype by introducing the RhoA inhibitor (C3) and the Rho kinase inhibitor (Y27632) to NBTII cells previously treated with Gleevec (**Figure 9A–C**, respectively). We found that adding either C3 (**Figure 9B**) or Y27632 (**Figure 9C**) to Gleevec-treated cells resulted in a significantly increased number of retraction fibers and more rounded nuclei compared to cells treated with Gleevec only (**Figure 9A**). In addition, both of these reagents reduced migration speed (**Figure 9D**) and persistence (**Figure 9E**). The nuclear aspect ratio and total retraction fiber length were calculated and compared to cells treated with Gleevec only or control cells (**Figure 9F–G**). C3 or Y27632 treated cells in the presence of Gleevec exhibit similar nuclear aspect ratios and retraction fiber parameters as the control group, suggesting that inhibition of RhoA/ROCK activity in Gleevec treated cells strongly rescues control cell phenotype. Compared with the significant change in nuclear aspect ratio and retraction fibers, the whole cell aspect and area ratios for the Gleevec + C3 treatment group or for the Gleevec + Y27632 group are partially rescued.(**Figure S4**). We also observed that while Gleevec + C3 or Gleevec + Y27632 treated cells produce extended lamellae these lamellae often fragmented during migration. Because C3 and Y27632 in effect rescue the control phenotype, these results indicate that RhoA and its downstream effector ROCK are required for the Gleevec- induced NBTII cell phenotype.

## Discussion

Here, we show that inhibition of Abl family kinase activity with Gleevec produced a rapid and remarkable change in cell morphology and migration in which cells spread out a thin, extended lamella and migrated faster and with more persistence with some similarities to fish and amphibian keratocyte migration [25], [27]. In addition, this rapidly spreading, very thin lamella is similar to the rapid and extensive, “pancake” spreading of fibroblasts derived from Abl null mice [15]. Associated with the Gleevec phenotype was an increase in RhoA activity, increased global cell adhesion strength, a pronounced change in adhesion patterns and an increase in total traction applied to the substrate.

### Regulatory mechanisms involved in the Gleevec-induced change in morphology and migration

#### Abl family kinases inhibit cell adhesion formation

Abl family kinases have been reported to be located at cell adhesions [7], [47]. They are correctly positioned to regulate the reorganization of the cytoskeleton at sites of membrane protrusion and at focal adhesions where integrins are engaged. In 10T1/2 fibroblasts, during the initial 20–30 minutes of fibronectin stimulation, when c-Abl activity is the highest, the nuclear pool of c-Abl re-localizes transiently to focal adhesions [7], [47]. This transient re-localization also occurs in NIH3T3 cells, where a fraction of the cellular Abl associates with the focal adhesion proteins, paxillin and Grb2 [48], [49]. Abl family kinases have also been reported to reduce initial cell attachment to the substrate. On fibronectin, fibroblasts derived from Abl-null mouse embryos spread faster than their wild-type counterparts, while restoration of Abl expression in the Abl-null fibroblasts reduced the rate of spreading [15]. Kain and Klemke provided evidence that Abl family kinases negatively regulate cell migration by uncoupling CAS-Crk complexes [13]. Li and Pendergast recently reported that Arg could disrupt CrkII-C3G complex formation to reduce β1-integrin related adhesion formation [17]. These reports indicate that Abl family kinases negatively regulate cell adhesion, thus supporting our observations that Abl family kinase inhibition results in a more adhesive and motile phenotype.

#### RhoA involvement in the Gleevec-induced phenotype

Concomitant with the adhesion increase induced by Gleevec treatment, there is an increase RhoA activity. Since Bradley and Koleske reported that Abl family kinases could function through the activation of p190RhoGAP to reduce RhoA activity [50], it is possible that the Gleevec action occurs by inhibition of the Abl-mediated activation of this RhoGAP. In any event, the increase in RhoA activity correlates with the increase in total traction force applied to the substrate; the spatial disposition of active myosin II indicates contractile activity parallel to the long axis of the cell and enhanced traction in the wings of the treated cell. Moreover, ROCK inhibition abrogates the Gleevec phenotype suggesting the pathway Abl inhibition→increase in RhoA activity→increase in ROCK activity→increase in pMLC→increase in contractility and traction.

It is important to note that Gleevec has been reported to have inhibitory effects on other signaling pathways involving PDGF-R and c-kit that also impact the cytoskeleton [51] and therefore, potentially, cell migration. Cells with inhibited PDGF-R or c-Kit pathways exhibit reductions in migration or membrane protrusions [46], [52], [53], [54] opposite to the effects reported here; this suggests that Gleevec inhibition of the c-kit and PDGF-R pathways is probably not the major factor for the profound NBT-II cell morphology transformation. Nevertheless, while Gleevec effects on Abl family kinase activity and cell adhesive behavior as well as on RhoA activity have been established [17], [50], it is well to keep in mind potential ‘off-target’ effects on other regulatory pathways.

### Changes in the adhesive behavior of Gleevec-treated NBTII cells

The D-shaped NBTII cells have a band of punctate dot-like adhesions in the vicinity of their leading edge that appear different from known adhesions in mesenchymal-type migrating cells. The area of these adhesions is quite small (∼0.10 µm^2^) compared to normal focal adhesions (∼1 µm^2^), and their turnover as estimated from observation of TIRFM movies is ∼1 min, compared to >5 min for focal adhesions. These punctate adhesions are similar in size to nascent focal adhesions [55], [56], [57]; but they rarely (<1%) matured to larger adhesions as many nascent adhesions do [57].

Often, an abundance of retraction fibers at the trailing edge of a cell is taken as evidence for strong adhesion in this region. However, at the rear of Gleevec-treated cells, in spite of greater global adhesion strength, there are fewer retraction fibers than in control cells. What might be the reason for this observation? A potential explanation is found in the fact that the trailing edge tractions of Gleevec-treated cells were significantly stronger than in control cells. These tractions may effectively break all adhesions in the rear of the cell, even those in that normally result in retraction fiber formation.

Our results taken as a whole indicate Abl family kinases play an important role in the regulation of cell adhesion and migration in that their inhibition produces a profound change in adhesions, morphology and cell migration. A fully integrated, quantitative view of inhibition of how these ubiquitous kinases produce these changes remains a challenge for the future.

## Materials and Methods

### Antibodies and Immunofluorescence

Antibodies: Integrin β-1 (CD29) blocking antibody anti-Mouse/Rat CD29 (HMβ1-1 BD. Biosciences Pharmingen, San Diego, CA), anti-α-Tubulin Antibody (#2144; Cell Signaling Technology, Beverly, MA), anti-phospho-myosin Light Chain II antibody (against Ser19, #3671, Cell Signaling Technology, Beverly, MA), anti-Rac1 antibody (#2465; Cell Signaling Technology, Beverly, MA), anti-Cdc42 antibody (#2462; Cell Signaling Technology, Beverly, MA), anti-RhoA antibody(sc-418; Santa Cruz Biotechnology, Santa Cruz, MA).

For immuno-staining, NBTII cells were fixed by using paraformaldehyde solution [4% (w/v) in PBS, pH 7.4] for 20 minutes at 25°C. Cells were then permeabilized with PBS containing 0.05% Triton-X-100 for 5 minutes at 25°C. Fluorescence labeling was carried out by treating with primary antibodies, washing with medium and then treating with fluorescent secondary antibodies followed by washing. Imaging was done as described below.

### Cell culture and transfection

NBT-II cells were acquired from the ATCC (Manassas, VA) and maintained in DMEM/F-12 medium (Gibco, Grand Island, NY) containing 10% FBS, 100 units/ml penicillin and 100 µg/ml streptomycin. The EGFP-Paxillin-β and EGFP-Vinculin construct were generated by subcloning DNA fragments expressing wild-type paxillin and wild type vinculin into a pEGFP-C1 vector (Clontech, Mountain View, CA). NBT-II cells were transfected using the Lipofectamine Plus transfection reagent (Invitrogen, Carlsbad, CA) according to the manufacturer's protocol. Cells stably expressing either EGFP-Paxillin-β or EGFP-Vinculin were obtained by sorting for EGFP positive cells after G418 selection in the UNC Flow Cytometry Facility.

### Cell migration, surface coating and drug treatment

For the experiments imaging the migration of NBT-II cells, glass bottom Petri dishes (35 mm) (MatTek) were coated by incubating with 10 µg/ml type I collagen (BD Biosciences, Bedford, MA) overnight at 4°C. NBT-II cells were treated with trypsin and resuspended in DMEM/F12 medium (GIBCO) containing 10% fetal bovine serum, plated at low density on the dishes, and cultured for 4-12 h in a CO_2_ incubator. Cells were incubated with either no inhibitor, or various concentrations of the 20 µM Abl family inhibitor Gleevec® (Novartis, Basel, Switzerland) by adding the inhibitor to culture media which was mixed by gently pipetting up and down. The cells were incubated for 30 minutes prior to imaging and imaging was performed in the continued presence of the inhibitor.

5 µM ROCK inhibitor(Y-27632, Sigma, MO) or 2 µg/ml of Rho inhibitor, C3 transferase (Cytoskeleton Inc., Denver, CO), was used to inhibit ROCK or Rho activity in Gleevec pre-treated NBTII cells for 30 minutes. 100 µg/ml RGD-containing peptide (Gly-Arg-Gly-Asp-Thr-Pro) (G5646, SIGMA), or 1 µg/ml anti-Mouse/Rat CD29 (Biosciences Pharmingen, CA) was used to block integrin related cell adhesion. Cell migration status was studied after one hour of incubation with these inhibitors.

### Assay for active RhoA GTPases

Active RhoA pulldown experiments were done as described previously [58]. For active RhoA pulldown cells were lysed in 300 µl 50 mM Tris, pH 7.4, 10 mM MgCl2, 500 mM NaCl, 1% Triton X-100, 0.1% SDS, 0.5% deozycholate, 1 mM PMSF, and 10 µg/ml each of aprotinin and leupeptin. Lysates were cleared by centrifugation at 14,000 g for 5 min. Supernatants were rotated for 20 minutes with 30-50 µg GST-RBD conjugated to glutathione–Sepharose beads (GE Healthcare). Beads were washed with in µl 50 mM Tris, pH 7.4, 10 mM MgCl2, 150 mM NaCl, 1% Triton X-100, 1 mM PMSF, and 10 µg/ml each of aprotinin and leupeptin. Active RhoA and total RhoA levels were analyzed by SDS-PAGE. Gel intensity results were quantified by analyzing inverted images using ImageJ. The signal from protein bands was measured after background subtraction and the intensity of each image was then normalized according to the average signal of loading control band.

### Cell imaging

Differential Interference Contrast, Total Internal Reflection Fluorescence (TIRF) and epi-fluoresence imaging were carried out on a dual-channel Olympus IX81 inverted microscope equipped with a 60×, oil immersion, 1.45 NA objective. Interference Reflection Microscopy imaging was performed using a 100×, oil immersion, 1.65 NA objective. Objective (×60) style TIRFM was performed on the Olympus IX81 with the Olympus TIR option. Images were captured using an air-cooled SensiCam QE CCD camera (Cooke Corp., Romulus, MI) driven by Metamorph (Molecular Devices/Meta Imaging, Downingtown, PA). Confocal imaging was performed with an inverted Olympus FV1000 equipped with a live cell chamber and a 60×1.42 N.A. oil immersion objective. Migration of single cells was tracked for durations between 5 minutes to 2 hours; time-lapse images were taken with the intervals of 1 second (for morphology changes), 5 seconds (for rapid adhesion turnover) or 60 seconds (for cell migration and long lifetime adhesion turnover), as indicated.

### Measurement of cell adhesion strength

A flow system designed to produce laminar shear stress on attached NBTII cells consisted of a flow cell, a dual syringe pump (Harvard Apparatus, Holliston, MA), 5% CO2 percolated media reservoir, and a pulse dampener (Cole Parmer Instrument Company, Chicago, IL) as previously described in detail [28], [29]. Briefly, a two-piece, top and bottom plate, anodized aluminum flow cell (12×7.5 cm) was constructed with plastic inlet and outlet tubes. The Nunc SlideFlask (Thermo) on which NBT-II cells were plated was placed in the middle of the flow cell bottom plate with a 0.27-mm thick silicone gasket placed underneath it. This brought the coverslip to the same height as the top of the bottom plate. Shear stress was calculated using the following equation:
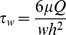
where μ is the viscosity of 0.0086 g.cm/s, Q is the flow rate in mL/s, w is the width of the flow channel (1.7 cm), and h is the height of the flow channel (0.027 cm). The applied shear stress ranged from 100 to 253 dynes/cm^2^.

Cells were cultured for 4 hours on 10 µg/ml pre-coated collagen Nunc SlideFlask (Thermo) substrates; and labeled with 1∶1000 Cell tracker orange (Invitrogen, Carlsbad, CA) for 10 minutes 30 minutes before flow experiments. All flow experiments were performed at 37°C for 1 minute with PBS containing calcium and magnesium. In each experiment, multiple (10–20) images were taken showing cells in different regions of the flow chamber, before and after shear stress was applied. The cell attached to substrate before and after shear stress were counted, expressed as the percent of adherent cells remaining, and averaged over multiple experiments. Data were reported as mean ± SEM.

### Traction force microscopy

Preparation of polyacrylamide substrates and experimental imaging has been described previously [42]. Briefly, the fabrication of polyacrylamide substrate involves following three steps: 1) Silanization the glass substrate with 0.5% silane for 20 minutes, 2) Use 0.5% glutaraldehyde treat previous substrate for 40 minutes, 3) polymerization with 6% polyacrylamide and 1% bis-acrylamide in 10 mM HEPES buffer with rhodamine-dextran beads and NHS-acrylate. Polymerization is initiated with the addition of 0.05 g/ml APS. The elastic modulus of our substrate was measured to be approximately 48 kPa [42]. Elastic substrates after polymerization were stored at 4°C in PBS.

Tractions were calculated using the method of Butler et al (2002) in which particle imaging velocimetry is employed to measure the displacement of small windows that contain a number of beads.

### Data quantification and calculation

The movement of individual cells was analyzed with Metamorph software and ImageJ. Statistical analysis was performed using unpaired student's t-tests. Statistical significance was indicated by * in the figure, and the p value was defined in each figure legend. One-way ANOVA with the Bonferroni post hoc test was used (as indicated in the figures) to compare the differences in NBT-II cell migration speed, persistence or other cellular morphology parameters, with values of P<0.05 sufficient to reject the null hypothesis for all analyses.

#### Speed and persistence of migration

To get the position of the cell, we manually tracked the geometric center of the cell nucleus. Although cell boundary morphology changed considerably during migration because of protrusion, the nucleus was usually centrosymmetric and could be employed as a marker for cell location. The cell position and cell migration speed was tracked and calculated using the ImageJ plugins: “Manual Tracking Plug-in” (http://rsbweb.nih.gov/ij/plugins/track/track.html). For cell migration persistence, we employed a conventional definition: the ratio of the net distanced traveled to the total distance traveled. The net distance traveled is the magnitude of the vector between the starting point and end point of the cell trajectory over an hour and the total distance traveled taken as the sum of net distances traveled over twelve 5 minutes intervals in that hour in order to capture the changes in direction that occur. Thus, the highest persistence would have a value of one, representing unidirectional migration.

#### Aspect ratio measurement and retraction fiber counting

The outline of the cell was manually obtained using Photoshop to trace the edge of each cell, based on DIC images. The dimension of the cell along the cell migration direction or perpendicular to cell migration direction was then measured from the cell outline with a Matlab program (The direction of cell migration was determined using ImageJ and manual tracking plugins as described above.). The aspect ratio was then calculated using cell dimension perpendicular to its migration direction divided by cell dimension along its migration direction. Retraction fibers could be counted manually because they were generally well-defined in the images.

#### Segmentation of adhesions in TIRF images

Segmentation of adhesions in TIRF images: Time-lapse TIRF images of EGFP-Paxillin were analyzed using previously published methods [59] [30]. First, the distribution of high-pass filtered pixel intensities was determined for each cell. Adhesions were segmented by selecting pixels at least two standard deviations away from the mean. Next, connected components labeling was used to identify the adhesions and any object less than two pixels in size was discarded. The average number of adhesions and the total area of the adhesions per image were calculated for each cell. T-tests were used to test for statistical differences between these measurements.

#### Lifetime of adhesions

ImageJ was used to measure changes in fluorescent intensity of individual adhesions over time in cells expressing fluorescent-tagged adhesion proteins. A perimeter was drawn around the punctate or wing adhesions at the point where intensity was a maximum; average pixel intensity was calculated from the pixel intensities within that perimeter as function of time. Background and photobleaching corrections were applied to obtain true intensities of the adhesions. Assembly and disassembly rates were plotted and calculated using Microsoft Excel (Microsoft Corporation) or Origin 6.1(OriginLab) using a previously published method (Huang et al., 2009) (Choi et al., 2008 [32].

#### Quantification of collective adhesion profile

Multiple lines (n = 4 for each cell, 12 cells in each group) were drawn along the cell migration direction from outside to the interior of the cell (line length = 200 pixels) with the center of the line is positioned manually at leading edge. The fluorescent signal along each line was measured using PlotProfile function in ImageJ. The EGFP-Paxillin fluorescence intensity in different cells was not uniform. In order to better compare the profile of collective adhesion intensities between control and Gleevec-treated cells, background (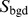) was subtracted from the raw fluorescence intensity profile (for each pixel S(n))and then these profiles were normalized. We used following formulae to get the normalized relative signal intensity (S_rsi-Line_(n)) for the n_th_ pixel (n = 1 to 200) along the line:
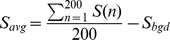


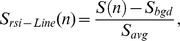
where 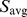 represents average pixel intensity averaged along the entire line.

## Supporting Information

Figure S1 **Increase in NBTII cell area after addition of Gleevec.** Addition of Gleevec at time = 0. Cell size is normalized to its size before Gleevec treatment.
(TIF)

Figure S2 **Optimizing the laminar shear stress flow system for cell adhesion strength measurements.** Panel A to F shows the adherent NBTII cells under laminar shear stress flow system with a 253 dynes/cm^2^ laminar force applied for 30 seconds. Cells are labeled with Cell Tracker Orange (Invitrogen). Images are same cells at different time points (before, 2 s, 5 s, 10 s, 20 s, 30 s) which are indicated at bottom right of each images. Arrows in each image point out the cells detached by the shear stress as a function of time of shear stress application. G) Left panel: Bar graph showing fraction of adherent cells retained after exposure to different laminar shear stresses for 1 min (N = 5 experiments; n = 11–20 images per N for 200 dynes/cm^2^; N = 1 experiment, n = 3 images per N for 100 dynes/cm^2^ and 253 dynes/cm^2^ groups). At 200 dynes/cm^2^, the difference between control and Gleevec-treated cells was significant (* p<0.01, by student's *t*-test). Error bars indicate standard deviations. Right panel: Determination of an approximate critical sheer stress for control and Gleevec-treated NBT-II cells. The critical shear stress at which 50% of the cells detached increased from 214 to 236 dynes/cm^2^ when cells were treated with Gleevec. NBT-II Cell detachment occurred predominantly at the level of integrin and other adhesion bonds to the matrix coated substratum as opposed to membrane rupture around the adhesion sites. Panel H) and I) show the fluorescent images of EGFP-Paxillin (H) and the actin cytoskeleton (I) visualized by Rhodamine-Phalloidin in the cell that remained attached after flow was applied. Cells that expressed EGFP-Paxillin were fixed after the flow experiment, stained with Rhodamine-Phalloidin and then imaged with 60× objective upright confocal microscope (Olympus FV1000) such that the optical section was close to substrate. J) A low power view (×20) showing cells after flow experiment with EGFP-Paxillin (green) and Phalloidin staining (Red). The image shows that almost all of the cells remained intact (marked with arrows); in fact, no fragments with adhesion proteins were observed in this and other views, suggesting that membrane failure was not the dominant mode of cell detachment. The average cell density before flow was about 10 cells/image. Bars in H and I are 20 µm and the bar in J is 100 µm.
(TIF)

Figure S3 **Cell adhesions in Gleevec-treated NBTII cells.** Panel A) and B) are interference reflection images and TIRF images for the same region in a fixed NBT-II cells. The dark dots (marked by arrows) and dark regions (marked by circles) in the interference reflection image were usually colocalized with the bright EGFP-Paxillin signal in TRIFM image, indicating these were cell-substrate adhesions. Panel C are the time-lapse images showing adhesion turnover at the leading edge of a Gleevec-treated NBTII cells. To better illustrate adhesion turnover, punctate adhesions at cell leading edge (at time 0) were marked with black line and then labeled with colored dots correspondingly. Adhesions at time 0, and after 30, 60, 90, 120, 150 and 180 seconds were shown. Colored dots indicate the previous adhesion is still remaining at this time. Most of the adhesions disassembled after 120 seconds. Panel D to G are representative TIRF images of EGFP-paxillin in Gleevec- treated NBTII cells, showing a rim of dense, punctate adhesions (adhesions in-between dotted lines) at the leading edge of the cells. Panel H is a temporal fluorescence intensity profile (see Materials and Methods) of EGFP-paxillin in a representative punctate adhesion at cell leading edge (a) or an adhesion at the side wings (b). Dotted lines I, II, III indicate the whole image fluorescent background, the cell leading edge fluorescent background, and the cell body fluorescent background respectively. The initial peak in the fluorescence intensity profile (marked by arrow) results from the formation of punctate adhesions. The lifetime is taken as time between liftoff from leading edge background (62) to when the intensity drops back to the cell body background (III). For the punctate adhesions at the leading edge the assembly and disassembly occurs quickly, with an average lifetime of ∼70 s (Panel I). By contrast, adhesions at the wings often gradually mature into strong and more stable adhesions with an average lifetime above 5 mins (Panel I). Scale bars in panels B and C are 5 µm, and in D, E, F, G are 20 µm. Data are mean ± standard deviations measured from 6–10 individual adhesions in 5–7 cells from independent experiments.
(TIF)

Figure S4 **RhoA/ROCK activity affects cell morphology.** Panel A) and B) are whole cell aspect ratio and cell area ratio. In each figure, four groups are control group, 20 uM Gleevec-treated group, 5 µM Y-27632+20 uM Gleevec-treated group, and 1 µg/ml C3+20 uM Gleevec-treated group, respectively. Error bars indicate standard deviations. At least 15 cells were measured for each group. The significance of the difference between control and other treated groups was evaluated by one-way ANOVA followed by Bonferroni's post hoc test, and marked by (*), p<0.05.
(TIF)

Movie S1 **Control NBT-II cell migration.** Time-lapse DIC microscopy recording of a control NBT-II cell migrating on collagen substrate (10 µg/ml). Movie demonstrates that NBT-II cells on collagen have medium-sized lamellae and lamellipodia, the multiple dynamic filopodia at the leading edge, and multiple retraction fibers at the rear end of the cell. Movie was recorded with 60× objective at 10 s intervals and plays back at 6 frames per second (fps).
(AVI)

Movie S2 **NBT-II cells in response to Gleevec treatment.** Time-lapse DIC microscopy recording of a NBT-II cell migrating on collagen substrate (10 µg/ml) and its response to Gleevec treatment (20 µM). Gleevec (20 µM) was added at time 00:00. Movie shows a quick transformation of cell morphology, including fast lamellipodia formation and changes of cell nucleus shape. Movie was recorded with 60× objective at 10 s intervals and plays back at 6 fps.
(AVI)

Movie S3 **Gleevec-treated NBT-II cell migration.** Time-lapse DIC microscopy recording of two Gleevec-treated (20 µM) NBT-II cells migrating on collagen substrate (10 µg/ml). Note that after Gleevec treatment NBT-II cells had an intact lamella facing the migration direction and markedly reduced numbers of both filopodia and retraction fibers. Movie was recorded with 60× objective at 10 s intervals and plays back at 6 fps.
(AVI)

Movie S4 **Special migration status and persistence in multiple Gleevec-treated NBT-II cell migration.** Time-lapse DIC microscopy recording of multiple Gleevec-treated (20 µM) NBT-II cells migrating on collagen substrate (10 µg/ml). NBT-II Cells were imaged immediate after Gleevec treatment. Note that NBT-II cells after Gleevec treatment have a big lamella facing the migration direction. The migration speed and persistence were significantly increased after Gleevec treatment. Movie was recorded with 20× objective at 5 min intervals and plays back at 3 fps.
(AVI)

Movie S5 **Adhesion in Control NBT-II cell imaged with interference reflection microscopy.** On the interference reflection images the areas of a cell that are closely apposed to the substrate appear dark indicating cell substrate adhesions. Time-lapse TIRFM of control NBT-II cells migrating on collagen substrate was recorded in interference reflection mode with 100× objective at 5 s intervals and plays back at 6 fps.
(AVI)

Movie S6 **Adhesion in Gleevec-treated NBT-II cell imaged with interference reflection microscopy.** Dark areas in interference reflection images are the regions where cell membrane is closely apposed to the substrate, indicating cell substrate adhesions. Movie demonstrates that Gleevec-treated cells formed a rim of punctate adhesions at their leading margin. Movie was recorded in interference reflection mode with 100× objective at 5 s intervals and plays back at 6 fps.
(AVI)

Movie S7 **Adhesion in Control NBT-II cell imaged with Total Internal Reflection Fluorescence Microscopy (TIRFM).** Time-lapse TIRF microscopy recording of control NBT-II cells migrating on collagen substrate; cells were transfected with EGFP-Paxillin. Movie was recorded in TIRFM mode with 60× objective at 15 s intervals and plays back at 4 fps.
(AVI)

Movie S8 **Adhesion in Gleevec treated NBT-II cell imaged with Total Internal Reflection Fluorescence Microscopy (TIRF).** Time-lapse TIRF microscopy recording of Gleevec-treated (20 µM) NBT-II cells migrating on collagen substrate; cells were transfected with EGFP-Paxillin. Note that Gleevec-treated cells have a rim of punctate adhesions at their leading margin. Movie was recorded in TIRFM mode with 60× objective at 15 s intervals and plays back at 4 fps.
(AVI)
