# Supplementary figures and images for: Gleevec, an Abl Family Inhibitor, Produces a Profound Change in Cell Shape and Migration
Source: PLoS One. 2013 Jan 2;8(1):e52233. doi: 10.1371/journal.pone.0052233 (PMC3534684; doi:10.1371/journal.pone.0052233)

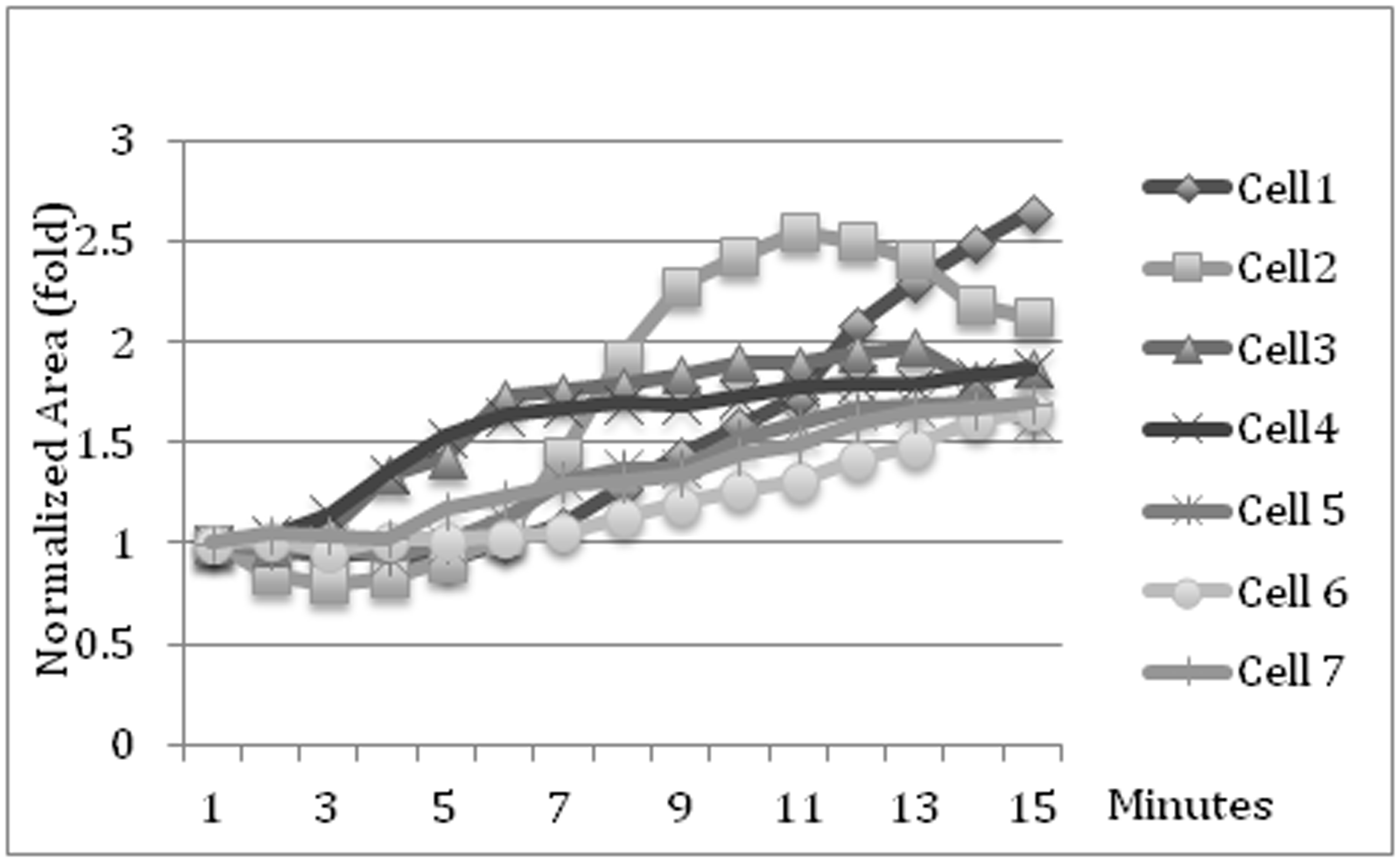

Supplement: Figure S1 — Increase in NBTII cell area after addition of Gleevec. Addition of Gleevec at time = 0. Cell size is normalized to its size before Gleevec treatment. (TIF) [file pone.0052233.s001.tif]

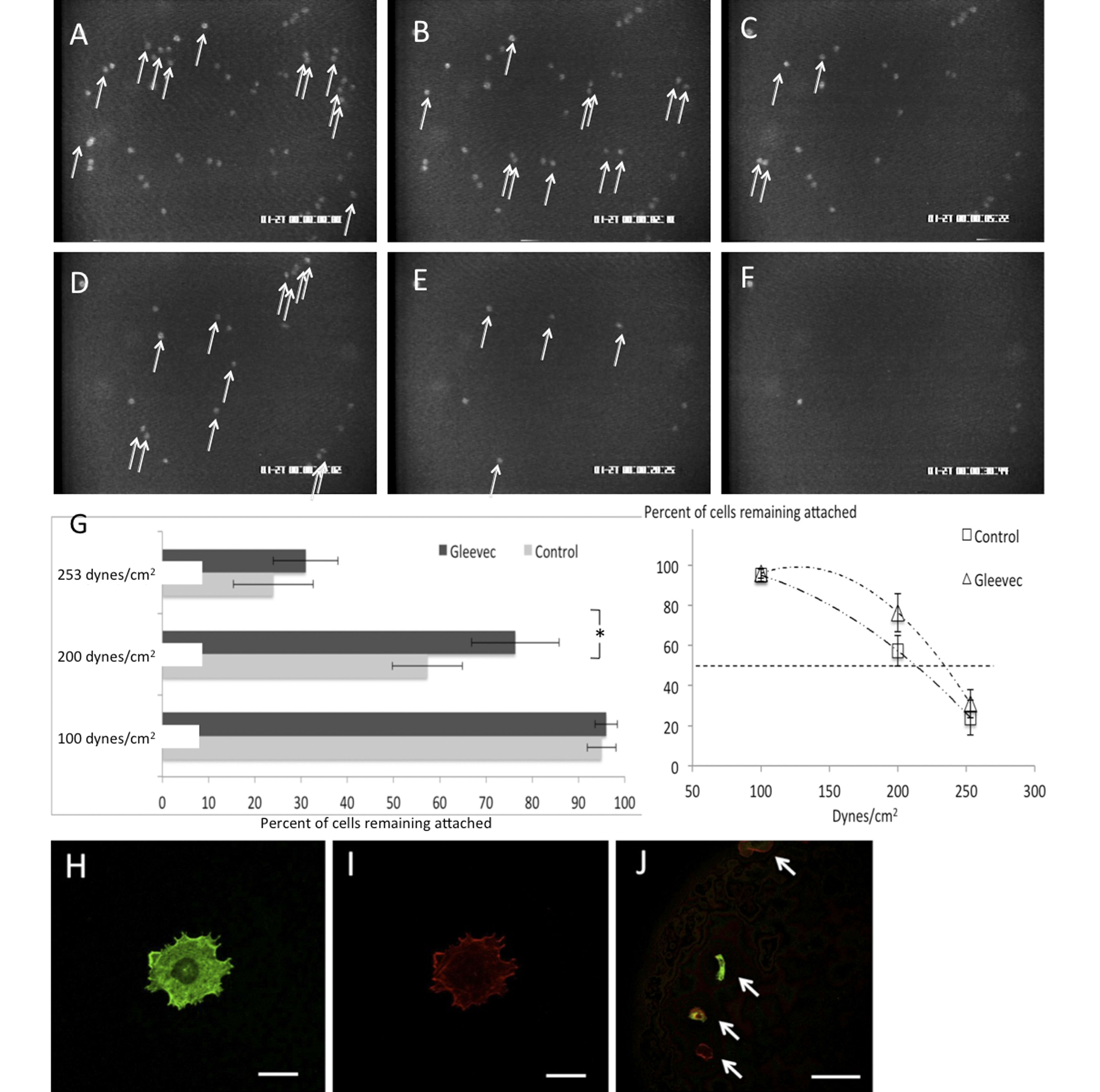

Supplement: Figure S2 — Optimizing the laminar shear stress flow system for cell adhesion strength measurements. Panel A to F shows the adherent NBTII cells under laminar shear stress flow system with a 253 dynes/cm2 laminar force applied for 30 seconds. Cells are labeled with Cell Tracker Orange (Invitrogen). Images are same cells at different time points (before, 2 s, 5 s, 10 s, 20 s, 30 s) which are indicated at bottom right of each images. Arrows in each image point out the cells detached by the shear stress as a function of time of shear stress application. G) Left panel: Bar graph showing fraction of adherent cells retained after exposure to different laminar shear stresses for 1 min (N = 5 experiments; n = 11–20 images per N for 200 dynes/cm2; N = 1 experiment, n = 3 images per N for 100 dynes/cm2 and 253 dynes/cm2 groups). At 200 dynes/cm2, the difference between control and Gleevec-treated cells was significant (* p<0.01, by student's t-test). Error bars indicate standard deviations. Right panel: Determination of an approximate critical sheer stress for control and Gleevec-treated NBT-II cells. The critical shear stress at which 50% of the cells detached increased from 214 to 236 dynes/cm2 when cells were treated with Gleevec. NBT-II Cell detachment occurred predominantly at the level of integrin and other adhesion bonds to the matrix coated substratum as opposed to membrane rupture around the adhesion sites. Panel H) and I) show the fluorescent images of EGFP-Paxillin (H) and the actin cytoskeleton (I) visualized by Rhodamine-Phalloidin in the cell that remained attached after flow was applied. Cells that expressed EGFP-Paxillin were fixed after the flow experiment, stained with Rhodamine-Phalloidin and then imaged with 60× objective upright confocal microscope (Olympus FV1000) such that the optical section was close to substrate. J) A low power view (×20) showing cells after flow experiment with EGFP-Paxillin (green) and Phalloidin staining (Red). The image shows that almost all [file pone.0052233.s002.tif]

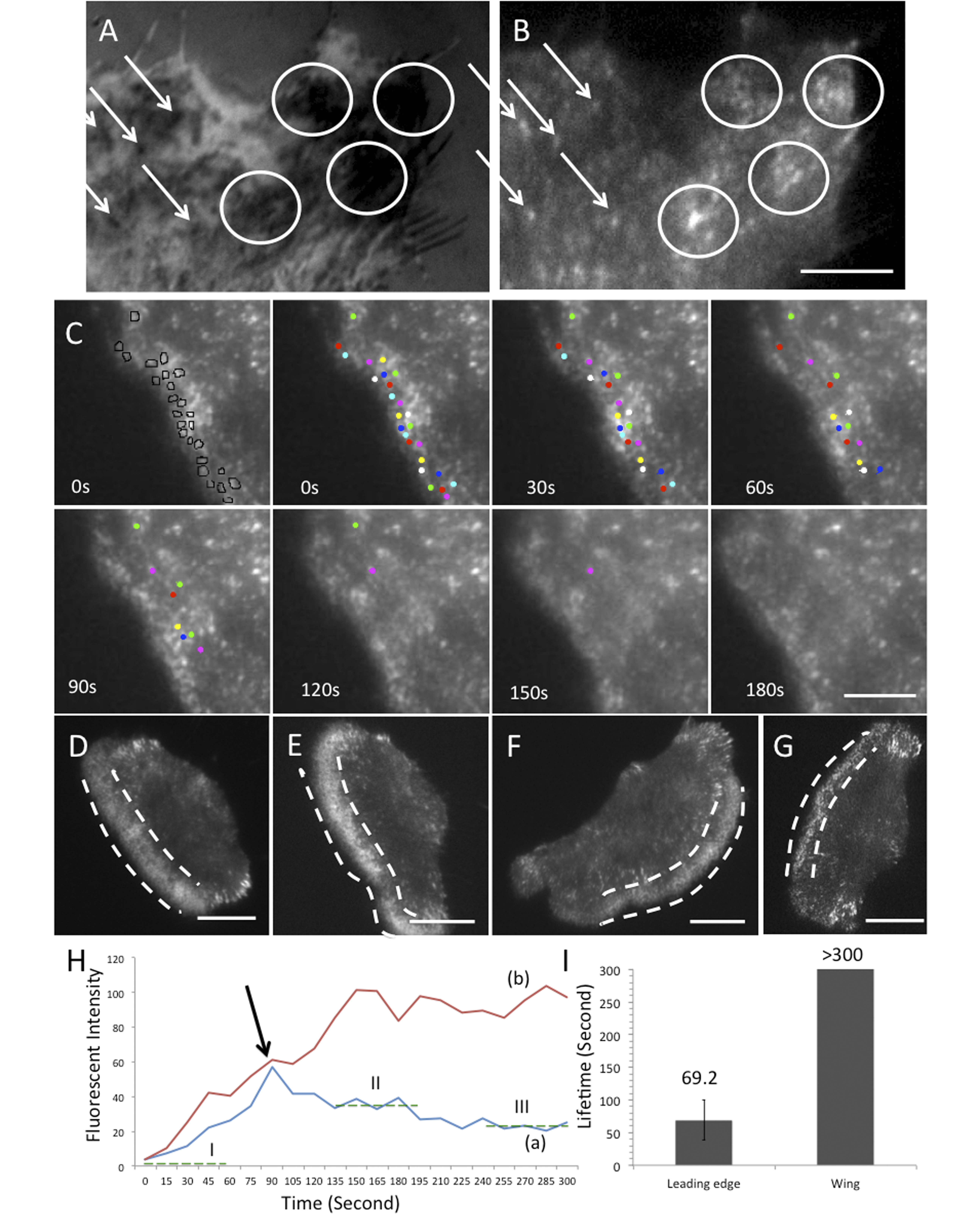

Supplement: Figure S3 — Cell adhesions in Gleevec-treated NBTII cells. Panel A) and B) are interference reflection images and TIRF images for the same region in a fixed NBT-II cells. The dark dots (marked by arrows) and dark regions (marked by circles) in the interference reflection image were usually colocalized with the bright EGFP-Paxillin signal in TRIFM image, indicating these were cell-substrate adhesions. Panel C are the time-lapse images showing adhesion turnover at the leading edge of a Gleevec-treated NBTII cells. To better illustrate adhesion turnover, punctate adhesions at cell leading edge (at time 0) were marked with black line and then labeled with colored dots correspondingly. Adhesions at time 0, and after 30, 60, 90, 120, 150 and 180 seconds were shown. Colored dots indicate the previous adhesion is still remaining at this time. Most of the adhesions disassembled after 120 seconds. Panel D to G are representative TIRF images of EGFP-paxillin in Gleevec- treated NBTII cells, showing a rim of dense, punctate adhesions (adhesions in-between dotted lines) at the leading edge of the cells. Panel H is a temporal fluorescence intensity profile (see Materials and Methods) of EGFP-paxillin in a representative punctate adhesion at cell leading edge (a) or an adhesion at the side wings (b). Dotted lines I, II, III indicate the whole image fluorescent background, the cell leading edge fluorescent background, and the cell body fluorescent background respectively. The initial peak in the fluorescence intensity profile (marked by arrow) results from the formation of punctate adhesions. The lifetime is taken as time between liftoff from leading edge background (62) to when the intensity drops back to the cell body background (III). For the punctate adhesions at the leading edge the assembly and disassembly occurs quickly, with an average lifetime of ∼70 s (Panel I). By contrast, adhesions at the wings often gradually mature into strong and more stable adhesions with an average lifetime a [file pone.0052233.s003.tif]

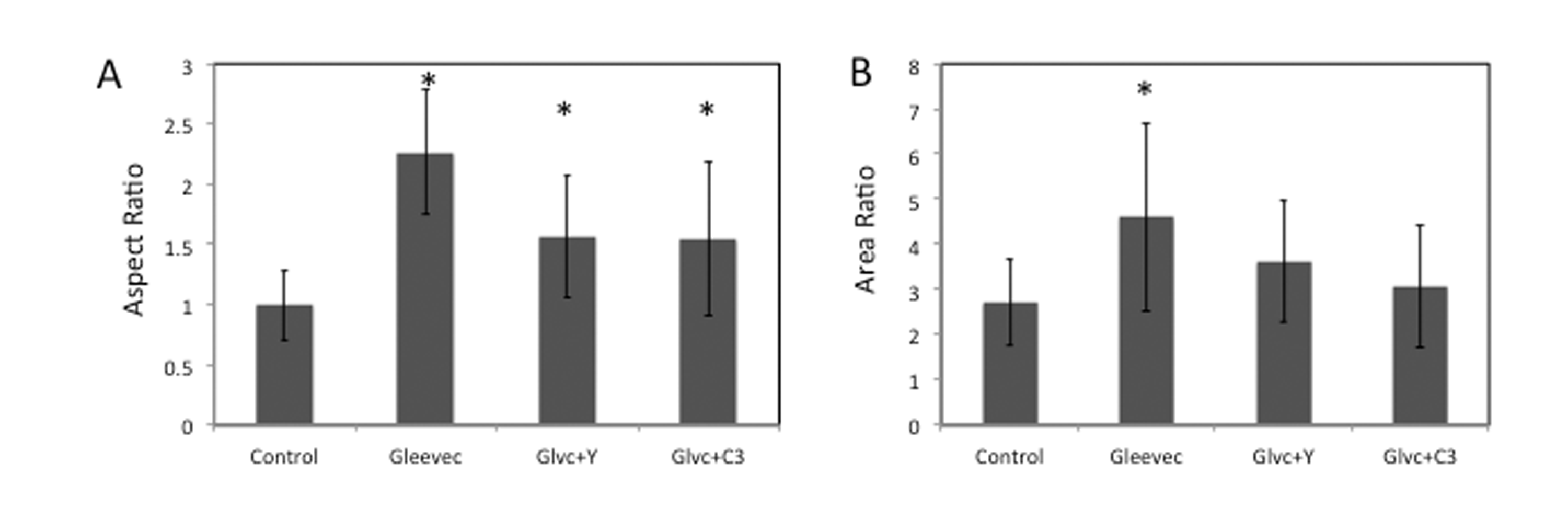

Supplement: Figure S4 — RhoA/ROCK activity affects cell morphology. Panel A) and B) are whole cell aspect ratio and cell area ratio. In each figure, four groups are control group, 20 uM Gleevec-treated group, 5 µM Y-27632+20 uM Gleevec-treated group, and 1 µg/ml C3+20 uM Gleevec-treated group, respectively. Error bars indicate standard deviations. At least 15 cells were measured for each group. The significance of the difference between control and other treated groups was evaluated by one-way ANOVA followed by Bonferroni's post hoc test, and marked by (*), p<0.05. (TIF) [file pone.0052233.s004.tif]
